# Effects of Salt-Reducing Alternatives on the Oral Processing Characteristics of Chickpea Nang

**DOI:** 10.3390/foods15050941

**Published:** 2026-03-07

**Authors:** Qian Wang, Ying Li, Sailimuhan Asimi

**Affiliations:** School of Public Health, Xinjiang Medical University, 567 Shangdebei Road, Urumqi 830017, China

**Keywords:** salt-reducing alternatives, nang, oral processing, mastication, saliva, food bolus

## Abstract

Salt reduction is an important strategy for healthy diets. Our previous study developed low-sodium chickpea nang (LCHN) using potassium chloride, wheat gluten, inulin and L-lysine. However, consumers also value taste. The impact of this reformulation on oral processing characteristics remains unclear. This study collected chewing samples from 12 volunteers at five mastication stages (0%, 25%, 50%, 75%, and 100%) of regular chickpea nang (CHN) and LCHN, measuring chewing parameters, bolus moisture content, saliva addition amount, and flow rate. Results indicated that LCHN had a significantly shorter swallowing time (24.22 ± 3.63 s vs. 27.84 ± 6.01 s, *p* < 0.05, Cohen’s d = 0.73), while the number of chews (Nc), chewing frequency (Fc), bolus moisture content, and saliva flow rate showed no inter-group differences across all mastication stages (*p* > 0.05). Bolus moisture content increased significantly with mastication progression in both groups (*p* < 0.05), whereas saliva addition amount and flow rate decreased significantly (*p* < 0.05). Additionally, higher chewing frequency correlated with increased saliva addition amount and reduced flow rate (*p* < 0.05). In CHN, the Nc positively correlated with chewing time (r = 0.452, *p* < 0.01) and frequency (r = 0.458, *p* < 0.01), whereas in LCHN it negatively correlated with time (r = −0.329, *p* < 0.05) and positively with frequency (r = 0.884, *p* < 0.01). These findings provide theoretical basis for low-sodium baked product development.

## 1. Introduction

As a typical traditional staple in northern China, nang is a high-temperature-baked cereal product with low moisture and a firm texture, making it easy to store and transport and suitable for the dry climate of northern China [1]. It is widely favored for its unique flavors, with onion, sesame and rose nang being popular varieties [2]. With rising health awareness, consumers increasingly prefer products combining health and good taste [3,4]. In recent years, studies on nang have mainly focused on new product development, formula optimization, processing technology, storage stability and its nutritional components, while its oral processing characteristics during consumption have rarely been reported [5].

Oral processing is the initial stage of digestion, involving physical breakdown and chemical digestion driven by mastication, salivary secretion, and tongue movement, which collectively form a swallowable food bolus [6]. This process dictates digestive efficiency and consumer acceptability, with chewing (physical reduction) [7] and saliva (enzymatic digestion and lubrication) [8,9] as core determinants. Studies have shown that food processing techniques can modulate oral processing behavior by altering texture [10]. Elucidating these mechanisms is therefore critical for guiding food formulation and sensory optimization. While oral processing research spans meat, dairy, and cereal products, the latter remain a primary focus due to their high consumption, providing a foundation for quality improvement [5].

Sodium chloride (table salt) serves as a fundamental ingredient in food formulations and a critical component in the production of flour products, playing a pivotal role throughout the entire production and consumption process. Beyond enhancing the savory and umami flavors of flour products, Na^+^ and Cl^−^ reduce electrostatic repulsion between glutenin subunits via electrostatic screening, promoting hydrophobic interactions and disulfide crosslinking to strengthen the gluten network, while hydration enhances dough water retention; additionally, high concentrations inhibit microbial growth by reducing water activity and exerting hyperosmotic stress [11]. These characteristics make sodium chloride an indispensable element in the industrial production of flour. However, excessive intake of sodium chloride can easily lead to health risks, which has become a global consensus. High-sodium diets have been proven to be a significant contributing factor to chronic diseases such as cardiovascular diseases, hypertension, and kidney diseases [12]. Therefore, dietary salt reduction has become a critical initiative for advancing public health worldwide. However, salt reduction in food products can easily lead to flavor deterioration, compromising the texture and mouthfeel of finished products and reducing consumer acceptance. Food salt reduction must not only meet consumers’ pursuit of healthy eating but also balance their dietary experience. To address this challenge, the food industry has developed sodium-free alternatives, flavor enhancers, and flavor peptides as salt substitutes.

These products compensate for quality deficiencies caused by reduced salt content through compound formulation or synergistic flavor effects [13]. In our previous study, potassium chloride was used to replace 30% sodium chloride, supplemented with wheat gluten, inulin and L-lysine to comprehensively improve the formula of chickpea nang, so as to establish a low-sodium flour product system [14]. Currently, research in the field of food and oral processing has focused on such salt-reduced alternatives, but most studies concentrate on their impact on flavor release pattern and sensory perception during processing. Currently, research in the field of food oral processing has been conducted on salt-reduced alternatives; however, existing studies have primarily focused on flavor release kinetics and sensory perception mechanisms (such as the effect of umami enhancers on saltiness perception in meat products) [15], while systematic investigations into key oral processing behavioral parameters (mastication efficiency and bolus formation) remain scarce. Furthermore, oral processing research on bakery products is seriously insufficient—the existing literature has mainly focused on static texture characteristics and sensory profiling of bread, lacking in-depth exploration of dynamic bolus property evolution during mastication. This study applied oral processing research methods to a salt-reduced bakery product system for the first time, using chickpea nang as the model, and revealed the influence mechanism of salt reduction alternatives on the entire oral processing procedure by monitoring mastication parameters, bolus moisture content, and salivary secretion, thereby filling the research gap in this field.

This study systematically analyzed the change patterns of chickpea nang and low-sodium chickpea nang during oral processing, and clarified the influence of salt substitution on oral processing characteristics. We hypothesize that with the progression of oral processing, the moisture content of boluses of both nang samples will increase significantly, while the amount of saliva added and saliva flow rate will decrease significantly, and this pattern will not be affected by salt substitution. The findings of this study aim to provide data support for clarifying the effects of low-sodium baked products on oral processing, thereby facilitating the development of low-sodium nang products to meet consumers’ dual demands for health and taste.

## 2. Materials and Methods

### 2.1. Basic Information on Chickpea Nang and Low-Sodium Chickpea Nang

Basic information on the chickpea nang and low-sodium chickpea nang is presented in Table 1. Prior to the oral processing experiment, both types of nang were prepared using the optimal formula derived from previous studies [16]. For the low-sodium chickpea nang, four added ingredients play important roles in compensating for salt reduction and improving product quality: wheat gluten and inulin can reduce the hardness of nang, L-lysine can improve the nutritional value of nang, and potassium chloride acts as a salt substitute to achieve the low-sodium target [14]. The two types of nang samples were cut into uniformly sized pieces (2 cm × 2 cm × 1 cm) according to their categories and batches. Each piece was weighed to be 3.50 ± 0.01 g, placed in a sealed bag, and labeled for subsequent use to minimize the impact of air, temperature, and humidity on the moisture content or texture of the sample.

### 2.2. Volunteer Screening

We recruited 12 volunteers interested in the experiment, including 7 males and 5 females, aged 22 to 26 years, with a normal body mass index (BMI 18.5–23.9). All volunteers were students from Xinjiang Medical University with a background in nutrition science. All recruited volunteers had no dietary preferences or smoking habits, and exhibited no allergic reactions to any food ingredients used in this study (wheat flour, chickpea flour, oil, water, yeast, salt, wheat gluten, inulin, L-lysine, potassium chloride), thereby minimizing the impact of allergies on salivary secretion. The volunteers had not received any dental treatment in the past six months, had no oral diseases or problems with mastication or swallowing, and did not take any medications that might affect oral mastication or salivary secretion. All volunteers signed a written informed consent form, completed the experiment successfully, and received financial compensation. This experiment was approved by the Ethics Committee of Xinjiang Medical University (14 March 2024), with the ethics approval number (XJYKDXR20230303030).

### 2.3. Experimental Procedure

This study was based on the method of Liu et al. [17] with some modifications. To ensure experimental consistency and minimize environmental and operational errors, all experiments were scheduled for 10:30 AM in a standard constant-temperature laboratory. Tables and chairs were fixed in place, and the food pellet box was labeled with a fixed code. The weighing balance (FA1204N, Shanghai Jinghai Instrument Co., Ltd., Shanghai, China) was calibrated before use to avoid interference from the experimental environment, equipment, or operational setup on the results. To address inter-individual variability, a two-level averaging strategy was employed: first, each sample was tested three times by each volunteer, and the individual mean was calculated as the representative value; second, subsequent inter-group comparisons were based on individual means, with the volunteer serving as the unit of analysis, effectively reducing individual variation. A total of four trials were performed, comprising two training sessions and two formal experiments. Prior to the experiments, volunteers underwent training to standardize operations (each session ≤ 30 min). Volunteers were instructed to chew and swallow samples, with chewing time (Tc) and chewing frequency recorded. Each sample was tested three times, and the average chewing time per volunteer was calculated. The total time from normal chewing to the swallowing point was recorded as 100% Tc, and then equally divided into four stages, 25% Tc, 50% Tc, 75% Tc, and 100% Tc for data collection. Prior to each training session and formal experiment, volunteers had to fast for 90 min to eliminate the effects of hunger or food digestion on chewing and swallowing.

During the formal experiment, volunteers were required to chew and spit out food bolus without collecting oral residues; oral rinsing was prohibited during chewing to prevent changes in the texture of the primary food bolus caused by drinking water or residual sample particles in the mouth. During the experimental procedure, food residues in the oral cavity were unavoidable. It was generally accepted that the collected food bolus could be used for sample analysis [18]. Therefore, the food boluses we collected did not include oral residues. Although the expectoration method differs behaviorally from natural swallowing and may introduce slight bias, this approach represents the standard method for obtaining stage-specific bolus samples in oral processing research. This study standardized operations through uniform training, and identical collection procedures were applied to both the control and low-sodium groups, ensuring that potential bias remained consistent across groups. After collecting all food boluses, volunteers were required to clean residual oral debris. To avoid fatigue induced by prolonged oral chewing that might affect experimental outcomes, the formal experiments for both types of nang were conducted in two separate sessions. In the formal experiment, all volunteers completed three parallel trials at each mastication stage. The total time for each volunteer should not have exceeded 20 min, with the individual chewing time for each sample not exceeding 1 min. Volunteers chewed two types of nang, producing at least 24 food boluses per person, resulting in a minimum of 288 boluses for the study. Samples were collected immediately after chewing for subsequent processing to prevent moisture evaporation from affecting experimental results.

### 2.4. Methods for Determining Oral Processing Parameters

Chewing parameters primarily include: chewing time, number of chews, and chewing frequency. The number of chews is the total number of occlusal contacts between the teeth during the natural chewing process, from food ingestion to swallowing. Chewing time denotes the duration consumed during this process. Chewing frequency (Formula (1)) is calculated as the ratio of the number of chews to the chewing time.

(1) Fc = Nc/Tc

The moisture content of the bolus refers to the saliva-containing food bolus expelled by volunteers during different oral processing stages based on their daily chewing habits. The moisture content of bolus (MC, Formula (2)) was determined by a direct drying method, where food boluses at different mastication stages were placed in an electric heating constant-temperature ventilation drying oven (DHG-9140A, Shanghai Qixin Scientific Instrument Co., Ltd., Shanghai, China) and dried at a constant temperature of 150 °C for 8 h. m_0_ is the initial weight of the sample, and m_1_ is the weight after drying.

(2) MC = (m_0_ − m_1_) × 100/m_0_

Saliva is primarily composed of water [19]. The amount of saliva added (SAn) at each mastication stage is calculated using Formula (5). Moisture content on the wet basis of a bolus (MC_wb_ bolus) represents the moisture content of the masticated bolus after drying, while moisture content on the wet basis of the food (MC_wb_ food) refers to the moisture content of the original food before mastication after drying. In this study, the wet basis moisture content (MC_wb_) was directly measured using Formula (2) and used for all saliva addition calculations. The dry basis moisture content (MC_db_) is defined by to Formula (3) for reference, but was not used in the data processing. The subscripts n and n − 1 are used to indicate different sample collection points.

(3) MC_db_ = MC/(1 − MC)

(4) SA(n) = MC_wb_ bolus(n) − MC_wb_ food

(5) ΔSA(n) = SA(n) − SA(n − 1)

Saliva flow rate (Sf, Formula (6)) is defined as the ratio of the amount of saliva added to chewing time.

(6) Sf(n) = SA(n)/Tc(n)

### 2.5. Statistical Analysis

Results were presented as mean ± standard deviation. The coefficient of variation (CV%) was calculated as (standard deviation/mean) × 100% to quantify inter-individual variability. K-means clustering analysis was performed to classify volunteers into distinct masticator subgroups based on salivary parameters (amount of saliva added and saliva flow rate) using standardized variables (scikit-learn library, Python 3.9). For data conforming to a normal distribution, one-way analysis of variance (ANOVA) followed by Duncan’s post hoc test was used for multiple comparisons, and Pearson correlation analysis was performed. For data not conforming to a normal distribution, the Kruskal–Wallis test was used, and Spearman correlation analysis was employed. An independent samples *t*-test was used to compare the two groups. Effect sizes were calculated as Cohen’s d. Between-cluster differences were assessed using an independent samples *t*-test or Mann–Whitney U test, as appropriate. All statistical analyses were performed using SPSS (version 26.0, Chicago, NY, USA) and Python 3.9. A *p* < 0.05 was considered statistically significant, while a *p* > 0.05 was deemed non-significant. Data visualization was completed using Origin 2022 (Origin Lab Inc., Northampton, MA, USA).

## 3. Results and Discussion

### 3.1. Changes in Chewing Parameters

The chewing parameters of the two types of nang during oral processing are shown in Table 2. At the swallowing point, there was a significant difference in chewing time between the two types of nang (*p* < 0.05). The chewing time for CHN was 27.84 ± 6.01 s, while that for LCHN was 24.22 ± 3.63 s, indicating that the chewing time of LCHN was shorter than that of CHN. This may be attributed to the impact of salt substitutes on the texture of CHN. Previous studies have found that LCHN made from salt-reducing alternatives has a lower hardness (72.77 N) and softer texture than CHN (146.52 N) [14], resulting in reduced chewing time from mastication to swallowing. There were no significant differences in the number of chews and chewing frequency of CHN during the four mastication stages (*p* > 0.05). There were no significant differences in the number of chews and chewing frequency of LCHN across the four mastication stages (*p* > 0.05). However, within the same mastication stage, significant differences were observed in both the number of chews and chewing frequency between the two types of nang (*p* < 0.05), with LCHN showing lower values at all four stages compared to CHN. Previous studies have also demonstrated that LCHN (116.52) exhibits lower chewiness than CHN (162.04), indicating that salt-reducing substitutes make LCHN more susceptible to damage during mastication [14]. Consequently, a lower number of chews and chewing frequency are required during the same chewing phase. In their study on the oral processing of rice and rice cakes, Choy et al. [20] also found that rice cakes required more chews than rice due to their higher hardness. Furthermore, during the 75–100% phase, the differences in chewing frequency and number between LCHN and CHN were most pronounced. This may be attributed to the impact of varying food textures on oral processing [21]. The loose texture of LCHN enables rapid saliva penetration and moistening of the decomposed food particles.

Throughout the oral processing, the average chewing frequency of volunteers chewing CHN was 1.12 Hz (1.08–1.14 Hz), and that for LCHN was 1.02 Hz (0.95–1.09 Hz). The difference in chewing frequency between the two types of nang was small. This may be due to the relatively stable chewing pattern when volunteers chewed similar food products. Compared with the chewing frequency of steamed grains (1.45–1.68 Hz) [17] reported in the previous literature, the average chewing frequencies of both types of nang in the present study were lower. This difference may be related to the textural properties of the food [22]. The nang used in this study was produced by oven baking, during which moisture was sufficiently evaporated, leading to higher hardness. As a result, the baked nang was easier to break down during mastication, thus requiring a lower chewing frequency. In contrast, steamed grains have higher moisture content, lower hardness, and greater stickiness [23], making them more difficult to break down during chewing, so a higher chewing frequency is needed to reach the swallowing threshold.

### 3.2. Analysis of Moisture Content of Bolus

**Figure 1:**
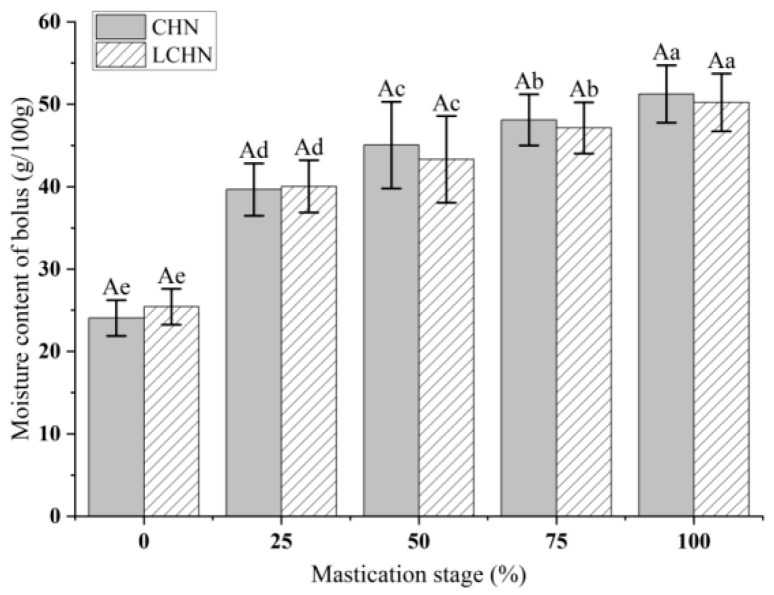
Changes in the moisture content of boluses of two kinds of nang at different mastication stages. Note: Different uppercase letters indicate significant differences between the two types of nang at the same mastication stage (*p* < 0.05). Different lowercase letters indicate significant differences between the same type of nang at different mastication stages (*p* < 0.05).

The results of the change in moisture content of boluses in the oral processing of the two kinds of nang are shown in Figure 1. The moisture contents of boluses of the two types of nang showed no significant difference during the same mastication stage (*p* > 0.05), indicating low variation in the moisture content of boluses at the same mastication stage. Tian et al. [24] demonstrated that the initial moisture content of white bread and whole-wheat bread significantly affected their water absorption rate and chewiness during oral processing, with bread with higher initial moisture content exhibiting a higher water absorption rate. Although this study observed the effect of initial moisture content differences on the oral processing process, it compared white bread with whole-wheat bread (which have significant differences in moisture content). In contrast, the initial moisture content of CHN and LCHN in the present study was similar, and the formulation difference was achieved primarily through salt substitution rather than moisture adjustment. Therefore, under the condition of similar initial moisture content, the type of food matrix had no significant impact on volunteers’ oral processing processes. This finding is consistent with the conclusion of Álvarez et al. [25], who observed that “initial moisture content had no significant correlation with chewing frequency” in a variety of solid foods. The study also revealed statistically significant differences (*p* < 0.05) in the moisture content of bolus across the five mastication stages in CHN, with an upward trend observed. The moisture content of bolus in LCHN exhibited statistically significant differences (*p* < 0.05) across the five mastication stages, with an upward trend. The results showed that the moisture content of boluses of the two kinds of nang increased significantly with the increase in the mastication stages, and the trend of change in the moisture content of boluses of the two kinds of nang was consistent.

In the initial stage of oral processing (0% stage), the moisture content of boluses of the two types of nang was the lowest, with the minimum moisture content of boluses of CHN being 24.07 ± 2.17 g/100 g and that of LCHN being 25.43 ± 2.50 g/100 g. At this stage, the food is only placed in the mouth, and the interior of the food has not yet mixed with saliva. At this time, the moisture content of the boluses is approximately equal to the original moisture content of the food. At this stage, the moisture content of LCHN’s boluses is slightly higher than that of CHN, likely due to the addition of water-absorbing accessories like inulin and wheat gluten in the LCHN formula [14]. Since CHN lacks these accessories, its natural moisture content is lower than LCHN’s, resulting in slightly lower moisture content of CHN’s initial bolus. During the initial phase of oral processing (0–25% stage), the moisture content of boluses of both types of nang increased more significantly than in other stages, which is consistent with the findings of Chen et al. [18]. At this stage, when food first enters the oral cavity, it is stimulated by contact with the tongue and teeth, as well as by taste and odor, leading to excessive salivary secretion that mixes with the food, resulting in a substantial increase in pellet weight. In the middle and late stages of oral processing (50–100% phase), the moisture content of boluses increased slowly for both types of nang. At the 100% stage, the maximum values were reached, with the moisture content of CHN’s bolus reaching 51.24 ± 3.49 g/100 g and that of LCHN’s bolus reaching 50.22 ± 2.11 g/100 g. By the time of swallowing, the moisture content of CHN’s bolus was slightly higher than that of LCHN’s bolus. The primary stage of increased moisture content of boluses in the food mass likely occurs during the initial phase of mastication. As the mastication stage progresses, the mucoprotein in saliva enhances the lubrication of the food bolus, interacts with food components and decomposes food structure, thereby increasing the moisture content of the food bolus particles [26].

### 3.3. Changes in Salivary Secretion

**Figure 2:**
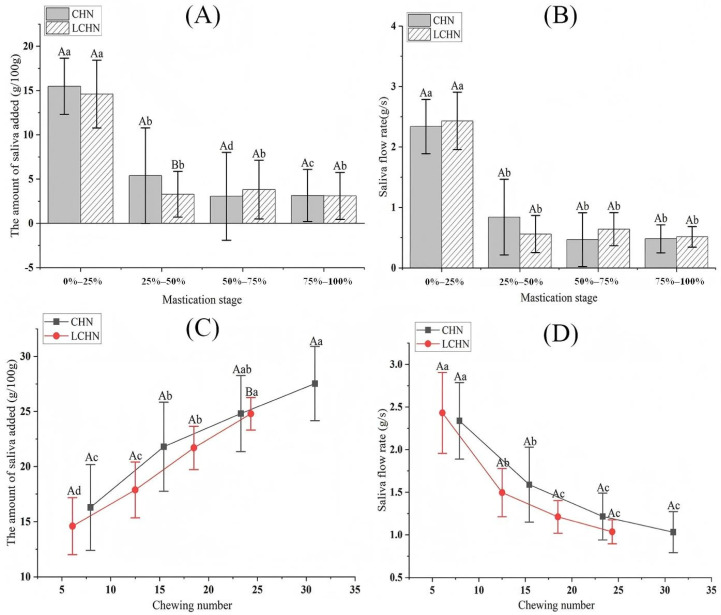
(**A**) The amount of saliva added to two types of nang at different mastication stages. (**B**) Saliva flow rate of two types of nang at different mastication stages. (**C**) Effect of the number of chews on the amount of saliva added for both types of nang. (**D**) Effect of the number of chews on saliva flow rate for both types of nang. The dots from left to right in Figures (**C**,**D**) represent 25%, 50%, 75%, and 100% of the oral processing stage, respectively. Note: Different uppercase letters indicate significant differences between the two types of nang at the same mastication stage (*p* < 0.05). Different lowercase letters indicate significant differences between the same type of nang at different mastication stages (*p* < 0.05).

Saliva plays a crucial role in the oral processing of food. The results of the amount of saliva added and saliva flow rate during different mastication stages for the two types of nang are shown in Figure 2A,B. There were statistically significant differences (*p* < 0.05) in the amount of saliva added and saliva flow rate between CHN and LCHN at all four mastication stages. As the mastication stage progressed, both the amount of saliva added and saliva flow rate of the two types of nang exhibited a downward trend. The amount of saliva added and saliva flow rate of the two types of nang were higher than the other three stages in the 0–25% phase, with both reaching their maximum values during this period. For CHN, the maximum amount of saliva added and saliva flow rate in the 0–25% phase were 15.48 ± 3.17 g/100 g and 2.34 ± 0.45 g/s, respectively. For LCHN, the maximum amount of saliva added and saliva flow rate in the 0–25% phase were 14.60 ± 3.83 g/100 g and 2.43 ± 0.47 g/s, respectively. The results showed that the initial stage of chewing (0–25%) was the main stage of salivary secretion and the stage of the largest saliva flow rate. The amount of saliva added and saliva flow rate of the two types of nang reached their minimum values at the 75–100% stage. For CHN, the minimum values of the amount of saliva added and saliva flow rate at the 75–100% stage were 3.15 ± 2.94 g/100 g and 0.48 ± 0.23 g/s, respectively. For LCHN, the minimum values of the amount of saliva added and saliva flow rate in the 75–100% were 3.10 ± 2.65 g/100 g and 0.51 ± 0.17 g/s respectively. This indicates that the secretion and flow rate of saliva in the later stages of chewing (75–100%) of nang products are significantly reduced. Furthermore, the difference in the amount of saliva added between the two types of nang during the 25–50% stage was statistically significant (*p* < 0.05), with CHN exhibiting slightly higher salivary secretion than LCHN. The difference in ingredients between the two types of nang may lead to distinct effects on salivary secretion. During the initial chewing phase, food is extensively broken down into particles of varying sizes, increasing the contact area with the oral cavity. The sodium chloride within these particles is gradually released into saliva and dispersed throughout the oral cavity through mechanical tongue movements [24]. Salt stimulation increases salivary secretion through the neural reflex arc [27,28]. LCHN uses salt-reducing alternatives to lower the sodium content of chickpea nang; with reduced salt, it irritates the salivary glands less, resulting in decreased saliva production.

The effects of the number of chews on the amount of saliva added and saliva flow rate are illustrated in Figure 2C,D. The results showed that the cumulative amount of saliva added to both kinds of nang increased with the increase in mastication time (*p* < 0.05), whereas the instantaneous saliva flow rate decreased (*p* < 0.05). At the swallowing point (100% stage), the cumulative amount of saliva added to CHN reached a maximum of 27.53 ± 3.37 g/100 g, at which point the saliva flow rate dropped to a minimum of 1.03 ± 0.24 g/s. Similarly, at the swallowing point, the cumulative amount of saliva added to LCHN reached a maximum of 24.80 ± 1.49 g/100 g, with the saliva flow rate dropping to a minimum of 1.04 ± 0.14 g/s. Liu et al. [17] similarly found that the cumulative amount of saliva added increased with the number of chews when individuals chewed different types of rice, whereas the saliva flow rate decreased with the increase in the number of chews. During oral processing, an increased numbers of chews not only generates food boluses composed of smaller particles but also enhances salivary secretion; high saliva flow rate can promote oral food processing [20,29]. The difference in the amount of saliva added between the two types of nang at the point of swallowing (100% stage) was statistically significant (*p* < 0.05), with CHN exhibiting slightly higher amounts of saliva added than LCHN.

### 3.4. Variations Among Volunteers

**Figure 3:**
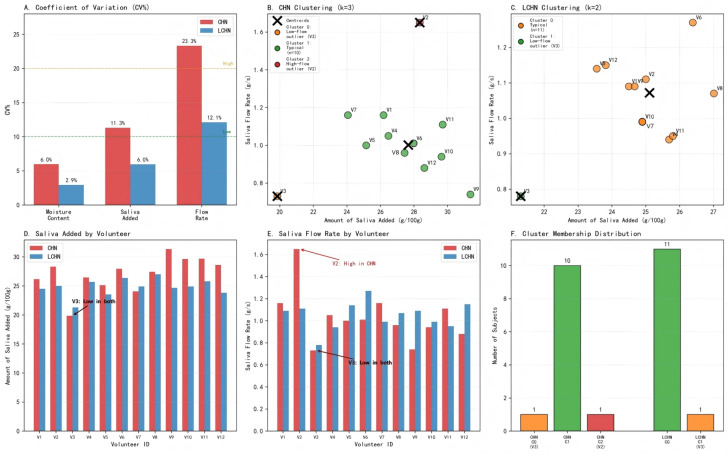
(**A**) Coefficient of variation across moisture content, saliva added, and flow rate; (**B**,**C**) K-means clustering identifying outlier volunteers (V2, V3) in CHN and LCHN conditions; (**D**,**E**) individual-level comparison of salivary parameters; (**F**) cluster membership distribution. Note: Volunteer numbers are represented by V1–V12.

**Figure 4:**
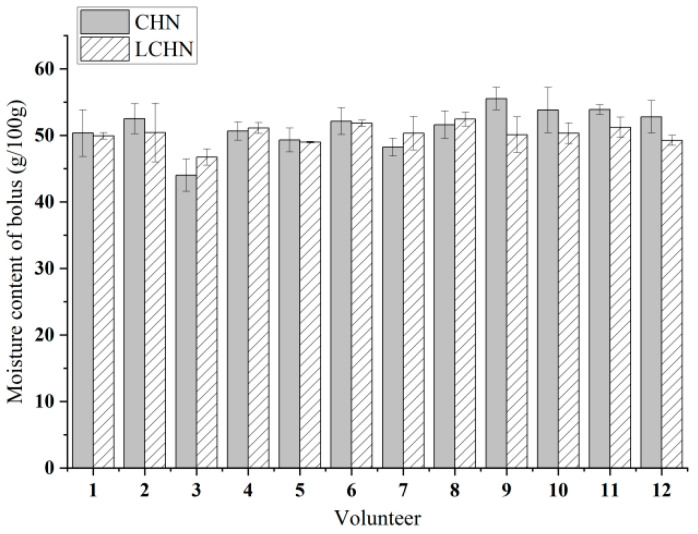
Individual variations in the moisture content of boluses.

Individual differences in volunteers are particularly important for food development and taste improvement. The inter-individual variability, analyzed by coefficient of variation (CV%) and K-means clustering, is shown in Figure 3. The individual differences in the moisture content of boluses at the swallowing point when volunteers chewed two types of nang are shown in Figure 4. The CV% for saliva flow rate was notably high (CHN: 23.3%; LCHN: 12.1%), indicating substantial inter-individual variability, whereas the moisture content of boluses showed low variation (CHN: 6.0%; LCHN: 2.9%) (Figure 3A). K-means clustering (k = 3) based on the amount of saliva added and saliva flow rate revealed distinct masticator subgroups for CHN: typical masticators (Cluster 1, n = 10), a low-flow outlier (Cluster 0, V3), and a high-flow outlier (Cluster 2, V2) (Figure 3B). For LCHN, two clusters were identified: typical masticators (Cluster 0, n = 11) and a low-flow outlier (Cluster 1, V3) (Figure 3C). Notably, V3 consistently clustered as an outlier across both samples, suggesting individual-specific physiological characteristics in salivary secretion.

Significant differences were observed in the moisture content of boluses, amount of saliva added, and saliva flow rate among the 12 volunteers (*p* < 0.05). These variations may be attributed to differences in chewing habits and physiological salivary function [30]. Joubert et al. [31] similarly reported significant inter-individual differences in saliva addition during bread chewing. Wada et al. [32] also found marked variations in chewing cycles among volunteers, with bolus moisture and saliva addition correlating with individual chewing patterns. Furthermore, individual differences in saliva flow rate and enzyme activity may influence starch hydrolysis efficiency [33], thereby affecting saliva addition at the swallowing point. The automated nature of chewing behavior [34] suggests that these individual patterns reflect consistent physiological traits rather than immediate sensory responses to food properties.

The results showed that there were slight differences in the moisture content of boluses, the amount of saliva added, and saliva flow rate at the swallowing point between CHN and LCHN. In CHN, the moisture content of boluses at the swallowing point was mainly concentrated at 50–53 g/100 g, the amount of saliva added at the swallowing point was mainly concentrated at 26–28 g/100 g, and the saliva flow rate at the swallowing point was mainly concentrated at 0.90–1.10 g/s. The maximum moisture content of boluses was 55.53 g/100 g, the maximum saliva added was 31.36 g/100 g, and the maximum saliva flow rate was 1.65 g/s. The minimum moisture content of boluses was 44.02 g/100 g, the minimum saliva added was 19.85 g/100 g, and the minimum saliva flow rate was 0.73 g/s. For LCHN, the moisture content of boluses at the swallowing point was mainly concentrated at 49–51 g/100 g, the amount of saliva added at the swallowing point was mainly concentrated at 24–26 g/100 g, and the saliva flow rate at the swallowing point was mainly concentrated at 0.90–1.00 g/s. The maximum moisture content of boluses was 52.44 g/100 g, the maximum saliva added was 27.01 g/100 g, and the maximum saliva flow rate was 1.27 g/s. The minimum moisture content of boluses was 46.74 g/100 g, the minimum saliva added was 21.31 g/100 g, and the minimum saliva flow rate was 0.78 g/s. This indicates that the moisture content of boluses and the amount of saliva added at the swallowing point vary among individuals [17].

### 3.5. Correlation Analysis

This section analyzes the correlations among parameters in each non-repetitive stage. The correlation results between the chewing parameters for the two types of nang and the moisture content of boluses, as well as saliva, are presented in Table 3. In CHN, the moisture content of boluses was positively correlated with the amount of saliva added (r = 1.000) and saliva flow rate (r = 0.958) (*p* < 0.01). It should be noted that the perfect correlation (r = 1.000) between bolus moisture content and saliva addition amount may reflect a mathematical dependence rather than a true physiological perfect correlation, which is a limitation of this study. This correlation indicated that the moisture content of boluses affected the amount of saliva added and saliva flow rate, and the amount of saliva added and saliva flow rate increased with the increase in the moisture content of boluses. The number of chews was significantly positively correlated with chewing time (r = 0.452) and chewing frequency (r = 0.458) (*p* < 0.01), while chewing time was significantly negatively correlated with chewing frequency (r = −0.403) (*p* < 0.01). These results indicate that the CHN chewing parameters are interrelated: an increase in chewing frequency is associated with a decrease in chewing time and a higher number of chews. The amount of saliva added was positively correlated with the saliva flow rate (r = 0.958) (*p* < 0.01), which indicated that the amount of saliva added of CHN was affected by the saliva flow rate, and the saliva flow rate increased with the increase in the amount of saliva added.

In LCHN, the moisture content of boluses showed a highly significant positive correlation with the amount of saliva added (r = 1.000) and saliva flow rate (r = 0.961) (*p* < 0.01), indicating that the moisture content of boluses affects the amount of saliva added and saliva flow rate, and both the amount of saliva added and saliva flow rate increase with an increase in the moisture content of boluses. The number of chews showed a significant negative correlation with chewing time (r = −0.329) (*p* < 0.05) and a highly significant positive correlation with chewing frequency (r = 0.884) (*p* < 0.01). Conversely, chewing time exhibited a highly significant negative correlation with chewing frequency (r = −0.533) (*p* < 0.01). These findings indicate mutual influence among chewing parameters in LCHN, where increased chewing frequency is associated with reduced chewing time and a larger number of chews. The amount of saliva added was positively correlated with the saliva flow rate (r = 0.961) (*p* < 0.01), which indicated that the amount of saliva added to LCHN was affected by the saliva flow rate, and the saliva flow rate increased with the increase in the amount of saliva added.

The correlation between chewing parameters and bolus moisture content in the two types of nang was generally consistent in the present study. Within the limitations of the current sample size, these results suggest that reducing salt content using salt-reducing alternatives had a minimal influence on chewing parameters, bolus moisture content, saliva added, and saliva flow rate. However, the correlation between the number of chews and chewing time was not consistent between the two samples. The textural differences observed may be attributed to the different formulations, and it appears that salt-reducing alternatives could influence the texture of chickpea nang, which might contribute to the shorter chewing time required to reach the swallowing point.

## 4. Conclusions

This study investigates the variation patterns of the oral processing of chickpea nang and low-sodium chickpea nang. The results showed that the swallowing time of LCHN was significantly lower than that of CHN. The differences in chewing frequency (0–25%, 25–50%), moisture content of boluses (all stages), amount of saliva added (0–25%, 50–75%, 75–100%) and saliva flow rate (all stages) between CHN and LCHN at the same mastication stage were all non-significant (*p* > 0.05), indicating that reducing the salt content of CHN by using salt-reducing alternatives did not significantly affect oral processing. In this study, a certain amount of variability in bolus moisture content, saliva addition amount, and saliva flow rate was observed among the 12 volunteers (affected by individual differences), and these results are not generalized to a wider consumer population. Correlation analysis revealed that the chewing parameter variables, moisture content of boluses, and saliva correlation, of the two types of nang were almost consistent, indicating that the reduction in salt content of CHN by salt-reducing alternatives had a minimal impact on its chewing parameters and saliva secretion. This study systematically analyzed the dynamic changes in CHN and LCHN during oral processing, which enriches the research on nang products in the field of oral processing. It provides a theoretical basis for the development of low-sodium products and the nutritional improvement of traditional baked flour products. We recommend the safe application of salt-reduction technologies in traditional nang and similar baked goods, and further optimization of formulas to compensate for deficiencies in flavor and texture, so as to promote the development of palatable, nutritious and healthy low-sodium baked products. The present study only focused on oral processing parameters, while other important aspects, including sensory perception (flavor compounds), physicochemical properties (taste compounds, texture characteristics, rheological properties, particle size distribution, and microstructure), and postprandial glucose response (satiety) during oral processing, were not investigated. Future studies will further explore the sensory perception, physicochemical properties, and postprandial glucose response to low-sodium baked flour products to provide a more comprehensive understanding.

## Figures and Tables

**Table 1:** Basic information on the two kinds of nang.

Name	Abbreviation	Raw Material	Sodium Content (mg Na/100 g)
Chickpea Nang	CHN	Wheat flour, Chickpea flour, Oil, Water, Yeast, Salt	177
Low-sodium chickpea Nang	LCHN	Wheat flour, Chickpea flour, Oil, Water, Yeast, Salt, Wheat gluten, Inulin, L-lysine, Potassium chloride	119

**Table 2:** Chewing parameters of two kinds of nang in different mastication stages.

Mastication Stage (%)	Number of Chews		Chewing Frequency (Hz)	
	CHN	LCHN	CHN	LCHN
0–25	7.46 ± 2.19 ^Aa^	6.08 ± 1.41 ^Ba^	1.14 ± 0.29 ^Aa^	1.04 ± 0.33 ^Aa^
25–50	7.23 ± 2.35 ^Aa^	6.42 ± 2.26 ^Ba^	1.11 ± 0.34 ^Aa^	1.09 ± 0.40 ^Aa^
50–75	7.42 ± 2.26 ^Aa^	6.00 ± 1.32 ^Ba^	1.13 ± 0.28 ^Aa^	1.01 ± 0.24 ^Ba^
75–100	7.04 ± 2.78 ^Aa^	5.58 ± 2.26 ^Ba^	1.08 ± 0.39 ^Aa^	0.95 ± 0.41 ^Ba^

Note: Different uppercase letters indicate significant differences between two types of nang at the same mastication stage (*p* < 0.05). Different lowercase letters indicate significant differences between the same type of nang at different mastication stages (*p* < 0.05).

**Table 3:** Analysis results of the correlation between chewing parameters and water content and saliva for two kinds of nang (Spearman correlation analysis).

	Chewing Number	Chewing Time	Chewing Frequency	The Amount of Saliva Added	Saliva Flow Rate
**CHN**					
Moisture content	0.011	−0.085	0.119	1.000 **	0.958 **
Number of chews		0.452 **	0.458 **	0.011	−0.025
Chewing time			−0.403 **	−0.085	−0.061
Chewing frequency				0.119	0.075
The amount of saliva added					0.958 **
**LCHN**					
Moisture content	0.025	0.000	−0.005	1.000 **	0.961 **
Number of chews		−0.329 *	0.884 **	0.025	0.087
Chewing time			−0.533 **	0.000	−0.219
Chewing frequency				−0.005	0.098
The amount of saliva added					0.961 **

Note: ** *p* < 0.01 (double-tailed), the correlation was significant. * *p* < 0.05 (double-tailed), the correlation was significant.

## Data Availability

The original contributions presented in this study are included in the article. Further inquiries can be directed to the corresponding author.
